# Uropathogen Distribution Across Successive Positive Urine Cultures Among Female Patients with Dysglycaemia

**DOI:** 10.3390/microorganisms14102126

**Published:** 2026-09-22

**Authors:** Sara Ali, Ansam Haidar, Farah Odeh, Sundus Abdishakur, Salma Elnour, Marwan Ismail, Praveen Kumar, Mahir Khalil Jallo

**Affiliations:** 1 Department of Medical Laboratory Sciences, College of Health Sciences, Gulf Medical University, Ajman P.O. Box 4184, United Arab Emiratesdr.salmaelnour@gmu.ac.ae (S.E.);; 2 Department of Physiotherapy, College of Health Sciences, Gulf Medical University, Ajman P.O. Box 4184, United Arab Emirates; 3 Department of Internal Medicine, Thumbay University Hospital, Ajman P.O. Box 4184, United Arab Emirates

**Keywords:** positive urine culture, dysglycaemia, *Escherichia coli*, extended-spectrum beta-lactamase, candiduria, urinary isolates, United Arab Emirates

## Abstract

Female patients with dysglycaemia may undergo repeated urine cultures, but longitudinal microbiological data from the Gulf are limited. We retrospectively examined successive positive urine cultures from 67 female patients attending a tertiary hospital in Ajman, United Arab Emirates, between May 2016 and September 2025. Organism identity, extended-spectrum beta-lactamase (ESBL) phenotype, HbA1c and culture dates were extracted from laboratory records. Twenty-eight participants (41.8%; 95% CI 30.5–53.7) had a second recorded positive culture and ten (14.9%; 95% CI 7.9–24.9) had a third; the median interval between the first and second cultures was 161 days (IQR 74–402). *Escherichia coli* was the most frequent organism at each culture. At the index culture, non-ESBL *E. coli* accounted for 38.8% of specimens, ESBL-producing *E. coli* for 25.4% and *Candida* species for 19.4%. Recovery of the same organism or phenotype category between the first and second cultures occurred most often with *E. coli* (17.9% non-ESBL; 14.3% ESBL-producing). Restricting the analysis to index cultures from 2023 to 2025 did not materially change the organism distribution. Because urinary symptoms and isolate-level susceptibility results were unavailable, positive cultures could not be classified as symptomatic infection, asymptomatic bacteriuria or candiduria, and treatment appropriateness could not be assessed. Prospective studies linking symptoms, susceptibility profiles and clinical outcomes are warranted.

## 1. Introduction

Urinary tract infection (UTI) is among the most common bacterial infections encountered in clinical practice, with women bearing most of the global burden [1,2]. Anatomical and physiological factors, including a short urethra and the proximity of the urethral meatus to the perineum, make ascending infection substantially more likely in women, and a majority will experience at least one episode during their lifetime [2,3]. A positive urine culture, however, is not synonymous with symptomatic UTI: interpretation requires compatible urinary symptoms and clinical context. This distinction is particularly important in retrospective laboratory datasets, in which bacteriuria or candiduria may be identified without sufficient information to establish clinical infection.

A subset of women experiences repeated urinary infections. Recurrent UTI is conventionally defined as two or more symptomatic episodes within six months or three or more within twelve months [2,3,4]. Recurrence may reflect relapse with a persisting strain or reinfection with a new strain; uropathogenic *E. coli* is capable of forming quiescent intracellular reservoirs within bladder epithelium that may seed later episodes, and the two mechanisms carry different implications for management. When symptoms and strain typing are unavailable, successive culture-positive events are more accurately described as repeated positive urine cultures rather than confirmed recurrent UTI.

Diabetes mellitus is associated with both incident and recurrent UTI [5,6,7]. Glycosuria may provide a growth substrate for uropathogens, autonomic neuropathy can impair complete bladder emptying, and neutrophil chemotaxis and cytokine responses may be altered in the diabetic state [6]. A systematic review conducted predominantly in women with type 2 diabetes reported recurrent UTI frequencies of 23.4–37%, with *E. coli* being the most frequent organism [5]. More recent retrospective work in diabetic populations has described a high burden of repeated episodes alongside rising antimicrobial resistance [7].

This question is particularly relevant in the Gulf. The Middle East and North Africa region has the highest comparative diabetes prevalence of any International Diabetes Federation region, and the United Arab Emirates is among the most affected countries [8,9]. In parallel, extended-spectrum beta-lactamase (ESBL)-producing Enterobacterales are well established across Gulf countries, with reported prevalence among urinary isolates of 25.2–31.7% [10]; a single-centre retrospective analysis of urine cultures in Riyadh reported ESBL production in 28.5% of *E. coli* and *Klebsiella* isolates, with complete resistance to third-generation cephalosporins and beta-lactam/beta-lactamase inhibitor combinations while carbapenems, aminoglycosides and fosfomycin retained activity [11]. Because a substantial proportion of ESBL-producing organisms in the region appear to be community-acquired rather than healthcare-associated [10], an ESBL phenotype cannot be assumed to signal prior hospital exposure. Describing organisms recovered over time from the same women may therefore provide useful local evidence for diagnostic and surveillance planning.

Existing regional studies have generally described uropathogen prevalence at a single time point. Less is known about organism distribution across successive positive cultures from the same women, or how often the same reported species is recovered. Laboratory data can address these microbiological questions even when the clinical record is insufficient to determine symptoms, treatment appropriateness or outcomes.

We therefore undertook a retrospective longitudinal laboratory study with three objectives: (i) to describe the frequency and timing of successive positive urine cultures among female patients with dysglycaemia; (ii) to characterise organism distribution at each recorded culture; and (iii) to quantify recovery of the same reported organism or resistance-phenotype category across successive cultures.

## 2. Materials and Methods

### 2.1. Study Design and Setting

This was a retrospective longitudinal laboratory-record study conducted at Thumbay University Hospital, Ajman, United Arab Emirates. The institution serves ambulatory and inpatient populations drawn largely from the northern emirates, and the source population is markedly multinational, spanning 60 recorded nationalities with no single group exceeding 17% of encounters. For institutional context only, the hospital recorded 1885 female encounters coded as ICD-10 N39.0 during 2024, presenting principally through Gynaecology (38.2%), Accident and Emergency (19.0%), Urology (15.6%) and Internal Medicine (12.3%). This encounter count is drawn from the hospital information system, which does not carry laboratory or glycaemic data and cannot be linked at patient level to the laboratory extract used in this study; it is therefore descriptive of the institution and is not the sampling frame for the present analysis. The study is reported in accordance with the STROBE statement for observational studies [12].

### 2.2. Participants and Eligibility

The sampling frame was a laboratory information system extract of urine cultures from female patients who met the operational definition of dysglycaemia given below, covering specimens received between May 2016 and September 2025. All consecutive eligible records within that window were extracted; no sampling or subsampling was applied, and no a priori sample size calculation was performed. Of 126 records screened, 59 were excluded because no organism was recorded at the index culture (57 with no organism entry and two documented as having no significant growth), leaving 67 participants for analysis. Each participant was then followed within the available record window to identify successive positive cultures. The participant flow is summarised in Table 1.

HbA1c was available for 65 of the 67 participants. Twelve of these values (18.5%) fell below the 6.5% diagnostic threshold for diabetes, ranging from 5.6% to 6.4%. Separately, the diagnosis, type and duration of diabetes could not be independently verified from the extracted records. Because the cohort therefore spans both the diabetes and the prediabetes ranges, and because diabetes could not be confirmed independently of the laboratory values, it is described throughout as female patients with dysglycaemia rather than as a diabetic cohort. For the two participants without an HbA1c, glycaemic status was established from an oral glucose tolerance test or fasting plasma glucose; both fell within the dysglycaemic range and neither met the biochemical threshold for diabetes.

Dysglycaemia was defined operationally, before analysis, as any recorded glycaemic measurement meeting American Diabetes Association criteria for either diabetes or prediabetes [13]: HbA1c ≥ 6.5%, fasting plasma glucose ≥ 126 mg/dL (7.0 mmol/L) or 2-h plasma glucose ≥ 200 mg/dL (11.1 mmol/L) during a 75-g oral glucose tolerance test for diabetes; and HbA1c 5.7–6.4%, fasting plasma glucose 100–125 mg/dL (5.6–6.9 mmol/L, impaired fasting glucose) or 2-h plasma glucose 140–199 mg/dL (7.8–11.0 mmol/L, impaired glucose tolerance) for prediabetes. HbA1c was the primary classifying test because it was recorded for 65 of the 67 participants; fasting plasma glucose and oral glucose tolerance testing were applied only where HbA1c was unavailable or fell below the prediabetes threshold. A recorded physician diagnosis of diabetes without a supporting glycaemic measurement was not by itself sufficient for inclusion.

Applying these criteria, all 67 participants met a threshold for dysglycaemia. Fifty-three (79.1%) had an HbA1c of at least 6.5%, within the diabetes range. Eleven (16.4%) had an HbA1c of 5.7–6.4%, within the prediabetes range. One participant with an HbA1c of 5.6% had a fasting plasma glucose of 110 mg/dL, meeting the criterion for impaired fasting glucose. Of the two participants without a recorded HbA1c, one had a 2-h plasma glucose of 173 mg/dL (impaired glucose tolerance) and one had a fasting plasma glucose of 112 mg/dL (impaired fasting glucose). The two fasting values of 110 and 112 mg/dL also exceed the higher World Health Organization threshold of 110 mg/dL for impaired fasting glucose, so neither classification depends on the choice of criterion. The distribution of glycaemic status is given in Table 2.

The cohort includes one participant aged 13 years. This record was retained because the eligibility criterion was a positive urine culture in a female patient with recorded dysglycaemia, without an age restriction, and because the institutional source population includes paediatric patients (11.7% of female N39.0 encounters during 2024 were in patients under 18 years). We note explicitly that discussion of adult-specific risk factors such as menopausal status and sodium–glucose cotransporter-2 (SGLT2) inhibitor exposure applies to the adult majority of the cohort; findings therefore principally reflect adults but are not restricted to adult women. A sensitivity analysis excluding this participant did not alter any reported proportion by more than one percentage point.

### 2.3. Definitions

**Significant growth.** Urine cultures were reported as showing significant growth by the clinical laboratory using standard quantitative criteria: growth of a single recognised uropathogen at a concentration of at least 10^5^ colony-forming units (CFU)/mL from a midstream clean-catch specimen, or at least 10^4^ CFU/mL where a single organism was recovered in pure culture from a symptomatic patient or from a catheter specimen. These thresholds correspond to routine laboratory practice at the study institution. Because the present study used the laboratory’s reported interpretation rather than re-deriving colony counts from primary plates, cultures are classified here exactly as reported in the laboratory information system.

**Mixed growth and possible contamination.** Cultures yielding three or more organism types, or yielding organisms consistent with periurethral or vaginal flora without a predominant uropathogen, were reported by the laboratory as mixed urethral flora and are retained in this analysis as a separate mixed-growth or possible-contamination category. Such cultures were not interpreted as identifying a uropathogen and were not counted towards organism-specific proportions. This category is reported transparently rather than excluded, because the distinction between contamination and genuine polymicrobial bacteriuria cannot be resolved retrospectively without a repeat specimen.

**Culture sequence.** The index positive culture was the first urine culture yielding significant growth for a participant within the study window. A subsequent positive culture was any later urine culture yielding significant growth in the same participant. The terms second and third indicate sequence within the available records and do not imply a uniform observation period.

**Interpretive limits.** Urinary symptoms, pyuria and clinical assessment were not available in the extracted records. Culture-positive events were therefore not assumed to represent symptomatic UTI, and we did not attempt to distinguish symptomatic infection from asymptomatic bacteriuria or asymptomatic candiduria. This distinction requires compatible symptoms, which were not recorded, and is discussed as a principal limitation. Recovery of the same organism or phenotype category was defined as recovery of the same reported organism and resistance-phenotype category at two cultures in the same participant. ESBL-producing *E. coli* is reported separately as a resistance phenotype but represents the same species as non-ESBL *E. coli*.

### 2.4. Microbiological Methods

Patients were instructed by trained staff to provide a midstream clean-catch urine specimen in a sterile container. Specimens were inoculated onto cystine–lactose–electrolyte-deficient agar using a calibrated loop and incubated aerobically at 37 °C for 18–24 h. Colony counts were performed and significance assessed against the criteria in Section 2.3.

Organism identification was performed by colonial morphology, Gram stain and conventional biochemical testing, supplemented by the automated DxM MicroScan WalkAway ID/AST system (Beckman Coulter, Brea, CA, USA) according to the manufacturer’s instructions. Routine quality control was undertaken under the laboratory’s Individualised Quality Control Plan using *Escherichia coli* ATCC 25922 and *Pseudomonas aeruginosa* ATCC 27853. Antimicrobial susceptibility testing was performed on the same automated platform and interpreted against Clinical and Laboratory Standards Institute (CLSI) M100 breakpoints of the edition current at the time of testing [14]; because specimens span several years, the breakpoint edition applied differed across the study period.

**Detection of resistance phenotypes.** Isolates showing reduced susceptibility to third- or fourth-generation cephalosporins underwent phenotypic confirmation of ESBL production by the modified double-disc synergy test. A 0.5 McFarland suspension of the test organism was swabbed onto Mueller–Hinton agar; an amoxicillin–clavulanate disc (20/10 µg) was placed centrally, with cefepime (30 µg) and ceftriaxone (30 µg) discs positioned 15 mm from the centre, and plates were read after 24 h at 37 °C. Enhancement or distortion of either cephalosporin inhibition zone towards the amoxicillin–clavulanate disc, producing the characteristic keyhole or champagne-cork morphology, was recorded as positive for ESBL production. *E. coli* ATCC 25922 and *Klebsiella pneumoniae* ATCC 700603 served as negative and positive controls, respectively. Methicillin resistance in *Staphylococcus aureus* was determined by cefoxitin screening on the automated platform and interpreted against the CLSI breakpoint current at the time of testing.

Yeast isolates were reported as *Candida* species without differentiation to species level; species-level identification was not undertaken at the study institution during the period covered and is therefore unavailable for this analysis.

**Availability of isolate-level results.** The variables retrievable from the laboratory information system for this study were organism identity and reported ESBL phenotype. Isolate-level screening zone diameters, confirmatory test readings and full susceptibility profiles were recorded in routine practice but could not be exported in the dataset extracted for this analysis and were therefore unavailable for independent re-evaluation. The methods described above characterise the laboratory workflow that generated the reported phenotypes; they do not imply that individual isolate results were re-examined by the investigators. Isolates were not retained and could not be retrieved for retesting.

### 2.5. Data Collection and Variables

Variables extracted included age at the index culture; HbA1c; fasting plasma glucose and oral glucose tolerance test (OGTT) results where recorded; organism or organisms recovered at each positive culture; reported ESBL phenotype; and the date of each positive culture. Fasting plasma glucose and OGTT values were present in the source records for a minority of participants and were used only to establish glycaemic status for the two participants lacking an HbA1c; they were not analysed as continuous variables. Data were extracted onto a standardised form and entered into a password-protected spreadsheet.

HbA1c appeared as a series of values within a single field for 15 participants. The value recorded closest to the index culture was used for the primary analysis, because glycaemic status at the time of the culture is the quantity of interest. A sensitivity analysis using instead the highest recorded value gave a mean of 8.09% (SD 2.07) against 8.04% (SD 2.10) for the primary analysis, and 41 rather than 40 participants at or above 7.0%; the choice of value therefore does not materially affect the reported summary.

### 2.6. Statistical Analysis

Continuous variables are summarised using the mean (SD) or median (IQR), as appropriate, and categorical variables as counts and percentages with Wilson 95% confidence intervals. Organism distributions across the index, second and third positive cultures are presented descriptively. No inferential comparison across culture sequence was performed, because later cultures were repeated observations from subsets of the original cohort and sparse organism categories precluded a reliable repeated-measures model. Recovery of the same organism or phenotype category was summarised using counts and percentages.

Analyses were performed on complete cases, with the denominator stated for each variable. No imputation was undertaken, as age served only to describe the cohort and was not used as an exposure, outcome or covariate. Two pre-specified sensitivity analyses are reported: one restricting the cohort to participants whose index culture fell between 2023 and 2025, and one excluding the single participant aged under 18 years. Analyses were performed in Python 3.12 with SciPy 1.17.

### 2.7. Ethical Considerations

Ethical approval was obtained from the Institutional Ethics and Research Committee of Gulf Medical University (Ref. No. IRB-COHS-STD-6-Jan-2025). The requirement for individual informed consent was waived because the study used retrospective, de-identified laboratory and clinical records. Data were accessible only to authorised study personnel and were handled in accordance with institutional policy and the Declaration of Helsinki. A fully de-identified dataset supporting all reported analyses is provided as Supplementary Materials.

## 3. Results

### 3.1. Participant Flow and Cohort Characteristics

Of 126 screened laboratory records, 59 were excluded because no organism was recorded at the index culture, leaving 67 female patients with an index positive urine culture (Table 1). These 67 participants contributed 105 positive urine cultures in total, of which 104 carried a retrievable specimen date. The dated specimens were weighted towards recent years: 73 (70.2%) were collected during 2024, 11 (10.6%) during 2023 and six (5.8%) during 2025, with the remaining 14 (13.5%) distributed across 2016–2022.

Age was recorded for 51 participants (76.1%), with a mean of 51.6 years (SD 17.8; median 49, range 13–85). HbA1c was recorded for 65 (97.0%), with a mean of 8.0% (SD 2.1; median 7.5, range 5.6–16.3); 40 of 65 (61.5%) had a value of at least 7.0%, while 12 (18.5%) had a value below 6.5%. Cohort characteristics are summarised in Table 2.

### 3.2. Frequency of Successive Positive Cultures

Of the 67 participants with an index positive culture, 28 (41.8%; 95% CI 30.5–53.7) had a second recorded positive culture. Ten participants had a third positive culture, corresponding to 35.7% (95% CI 20.1–54.2) of those with a second culture and 14.9% (95% CI 7.9–24.9) of the index cohort. The median interval between the first and second cultures was 161 days (IQR 74–402; available for 27 of 28), and that between the second and third was 185 days (IQR 86–392; available for 9 of 10). Fifteen of 27 participants (55.6%) with a measurable first-to-second interval had the second positive culture within six months.

In the pre-specified sensitivity analysis restricted to the 58 participants whose index culture fell between 2023 and 2025, 20 (34.5%; 95% CI 23.2–47.2) had a second positive culture and five (8.6%; 95% CI 3.4–17.9) a third. These proportions are lower than in the full cohort, which is expected because the shorter available record window reduces the opportunity for a further culture to be captured, and reinforces that these figures reflect record availability rather than a rate over a common observation period.

### 3.3. Organism Distribution Across Positive Cultures

Across the index, second and third positive cultures, *E. coli* remained the most frequently recovered organism. Non-ESBL *E. coli* accounted for 26 of 67 index specimens (38.8%; 95% CI 27.7–51.0), 10 of 28 second-culture specimens (35.7%; 95% CI 20.1–54.2) and five of 10 third-culture specimens (50.0%; 95% CI 22.3–77.7). ESBL-producing *E. coli* accounted for 17 of 67 index specimens (25.4%; 95% CI 16.4–36.8), while *Candida* species accounted for 13 of 67 (19.4%; 95% CI 11.5–30.4). When non-ESBL and ESBL-producing isolates were combined, *E. coli* represented 43 of 67 index specimens (64.2%; 95% CI 52.3–74.9). Estimates for later cultures were imprecise because only 28 and 10 participants contributed second and third cultures, respectively (Table 3; Figure 1).

In the sensitivity analysis restricted to index cultures from 2023 to 2025 (*n* = 58), the organism distribution was essentially unchanged: non-ESBL *E. coli* 21 (36.2%), ESBL-producing *E. coli* 15 (25.9%) and *Candida* species 12 (20.7%). The organism findings are therefore not driven by the small number of older specimens.

### 3.4. Recovery of the Same Organism or Phenotype Category

Recovery of the same reported organism or resistance-phenotype category between consecutive cultures was most frequent for *E. coli*. Between the index and second cultures, non-ESBL *E. coli* was recovered on both occasions in five of 28 participants (17.9%), ESBL-producing *E. coli* in four of 28 (14.3%) and *Candida* species in two of 28 (7.1%).

Among the 10 participants who contributed a third culture, non-ESBL *E. coli* was recovered at both the second and third cultures in one (10.0%) and ESBL-producing *E. coli* in two (20.0%). Comparing the index and third cultures, non-ESBL *E. coli* was recovered on both occasions in two participants (20.0%) and ESBL-producing *E. coli* in three (30.0%); *Candida* species were not recovered at both the index and third cultures in any participant. These later comparisons rest on 10 participants and are correspondingly imprecise. Because strain typing was not performed, none of these findings can distinguish persistence or relapse from reinfection with another strain of the same species (Table 4).

## 4. Discussion

In this retrospective laboratory cohort of 67 female patients with dysglycaemia, 41.8% had a second and 14.9% had a third recorded positive urine culture. *E. coli* predominated across culture sequence; when non-ESBL and ESBL-producing isolates were combined, it accounted for 64.2% of index specimens. ESBL-producing *E. coli* represented approximately one quarter of index specimens, and *Candida* species nearly one fifth.

### 4.1. Successive Positive Cultures in Regional and International Context

The observed 41.8% frequency of a second recorded positive culture is higher than the 23.4–37% recurrent-UTI range reported in a systematic review of women with type 2 diabetes [5], but these measures are not directly equivalent. Our denominator comprised women already selected for an index positive culture, observation periods differed, and symptom data were unavailable. The estimate is therefore neither a population incidence nor a clinical recurrence rate. That the corresponding proportion fell to 34.5% when the cohort was restricted to index cultures from 2023 to 2025 illustrates the point directly: the figure is a function of how long each participant’s record was available for inspection. It instead identifies a subgroup with repeated culture-positive events requiring prospective investigation under a common follow-up period and a symptom-based case definition.

### 4.2. Organism Spectrum and the ESBL Burden

The predominance of *E. coli* is consistent with the established microbiology of UTI in women with diabetes [5,6]. ESBL-producing *E. coli* represented 25.4% of index specimens, consistent with the substantial ESBL burden described in Gulf and regional studies [10,11], and unchanged at 25.9% when the analysis was restricted to recent years. This finding is clinically relevant because an ESBL phenotype can restrict therapeutic options; however, isolate-level susceptibility results were unavailable, and the activity of individual agents cannot be inferred from these data. Future surveillance should link organism identification, resistance phenotype and complete susceptibility profiles.

The descriptive distribution of major organism groups appeared broadly similar across culture sequence, but this should not be interpreted as evidence of equivalence. Only 28 and 10 participants contributed second and third cultures, respectively, and repeated observations from the same women precluded a valid independent-samples comparison. Recovery of the same reported organism or phenotype category, particularly for *E. coli*, was observed at every pairwise comparison; strain-level typing would be required to distinguish persistence or relapse from reinfection.

### 4.3. Candida Species and Interpretive Uncertainty

*Candida* species were recovered from 19.4% of index specimens. Without urinary symptoms, pyuria, repeat confirmatory sampling or clinical assessment, this finding cannot be classified as symptomatic *Candida* UTI and may represent candiduria or colonisation. Guidance discourages routine treatment of asymptomatic candiduria except in selected high-risk circumstances [15]. Diabetes-related glycosuria is biologically relevant, and sodium–glucose cotransporter-2 (SGLT2) inhibitor exposure, which acts by inducing glycosuria, may also influence genitourinary Candida risk [16]; glucose-lowering therapy was not recorded in the present dataset. Prospective studies should capture symptoms, pyuria, pregnancy or procedural status, catheterisation and SGLT2 inhibitor exposure before assigning clinical significance to *Candida* recovery.

### 4.4. Strengths and Limitations

The principal strength of this study is its longitudinal laboratory perspective: it follows the same participants across successive positive urine cultures rather than presenting only a single cross-sectional snapshot. This permits direct description of organism distribution and of recovery of the same reported organism or phenotype category across culture sequence, in an emirate for which published longitudinal microbiology is limited, and in a markedly multinational source population.

Several limitations materially constrain interpretation. First, urinary symptoms were unavailable; positive cultures cannot be assumed to represent symptomatic UTI, and asymptomatic bacteriuria or candiduria cannot be separated from clinical infection. Second, specimens span May 2016 to September 2025 and observation periods differed among participants, so the proportion with another positive culture is not a rate over a common interval. Sixteen participants contributed only one recorded specimen in 2024 and had no recorded age or subsequent culture in the available dataset; it was not possible to determine whether this reflected absence of another culture-positive event, incomplete follow-up, or subsequent care outside the study institution, and absence of a subsequent culture should therefore not be interpreted as absence of recurrence. Third, glycaemic classification was imprecise because diabetes diagnosis, type, duration and treatment were not consistently available. Fourth, age was missing for 23.9% of participants and one participant was 13 years old. Fifth, isolate-level susceptibility results were unavailable, preventing analysis of resistance profiles beyond the recorded ESBL phenotype, and isolates were not retained for retesting. Sixth, strain typing was not performed, so persistence or relapse cannot be distinguished from reinfection. Seventh, only 28 and 10 participants contributed second and third cultures, respectively, resulting in imprecise estimates and preventing a reliable repeated-measures comparison. Finally, menopausal status, pregnancy, catheterisation, renal function, prior antimicrobial exposure and glucose-lowering therapy were not captured.

Source-record consistency was an additional limitation. Several variables were recorded in free text with inconsistent spelling and spacing. Nine negative entries in the OGTT field were interpreted as corrupted date serials and treated as missing. HbA1c appeared as multiple values within a single field in 15 records, addressed as described in Section 2.5. These data-quality constraints reinforce the need for a structured collection instrument and predefined longitudinal sampling in future work.

### 4.5. Implications and Future Directions

The ESBL burden and repeated recovery of *E. coli* support laboratory surveillance that links organism identity, complete susceptibility profiles and prior culture history. Culture findings must be interpreted alongside symptoms and patient factors rather than in isolation. A prospective cohort should apply a symptom-based case definition and a common follow-up period, capture diabetes diagnosis and duration, HbA1c, pregnancy, menopausal status, catheterisation, renal function, and SGLT2 inhibitor and antimicrobial exposure, and measure clinical outcomes. Molecular typing would permit relapse to be distinguished from reinfection, and retention of isolates would allow susceptibility profiles to be linked to culture sequence.

## 5. Conclusions

Among female patients with dysglycaemia and an index positive urine culture at a tertiary hospital in Ajman, *E. coli* was the predominant organism across successive cultures. ESBL-producing *E. coli* accounted for approximately one quarter and *Candida* species for nearly one fifth of index specimens, and these proportions were unchanged when the analysis was restricted to recent years. Later-culture estimates were limited by small sample sizes and non-uniform follow-up. Because symptoms, isolate-level susceptibility profiles and treatment outcomes were unavailable, the results should be interpreted as laboratory recovery patterns rather than as confirmed symptomatic infections or as evidence of treatment quality. Prospective, clinically linked microbiological surveillance is required to determine recurrence, resistance and outcomes.

## Figures and Tables

**Figure 1:**
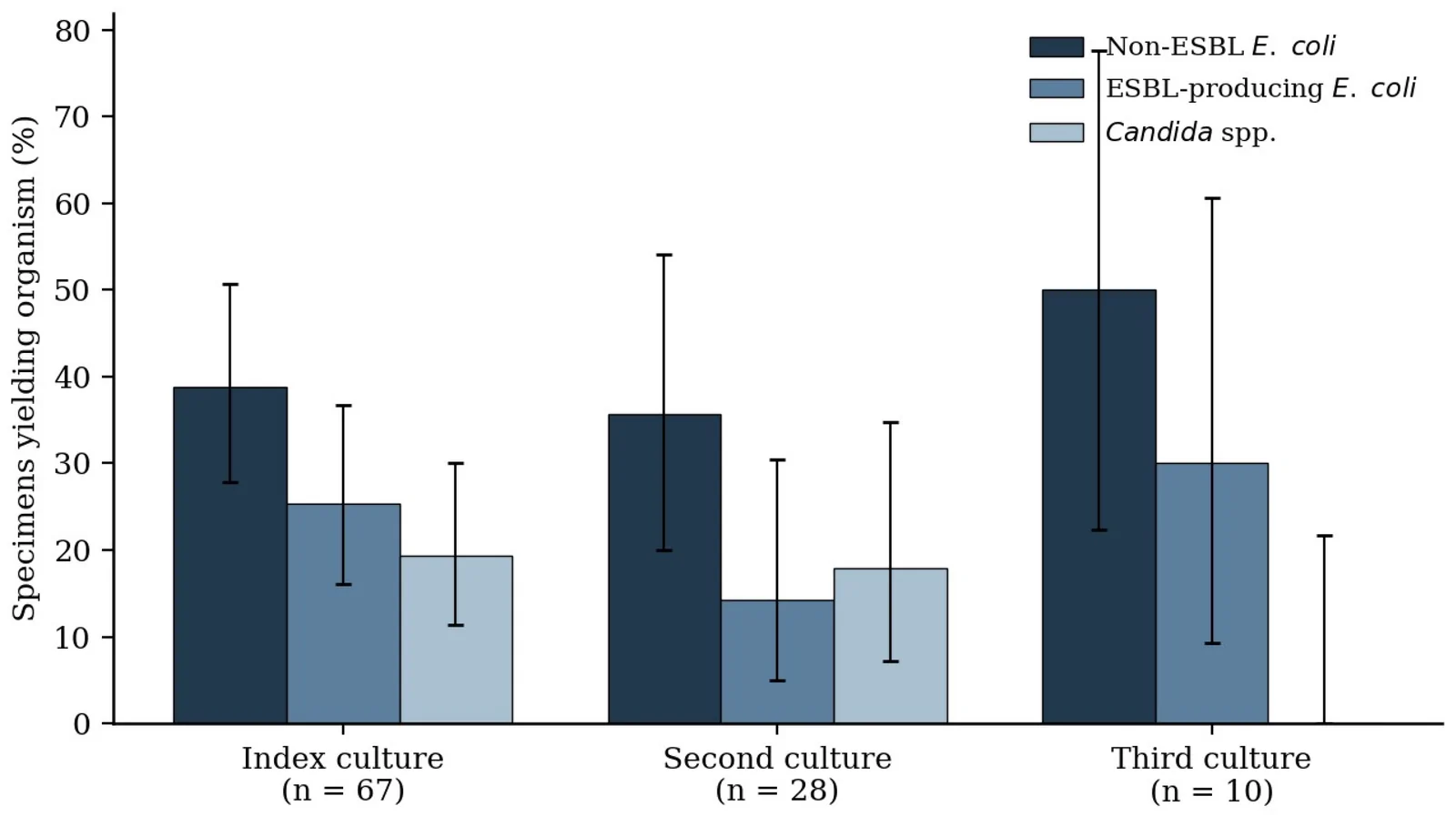
Proportions of urinary specimens yielding non-ESBL *Escherichia coli*, ESBL-producing *E. coli* and *Candida* species at the index (*n* = 67), second (*n* = 28) and third (*n* = 10) positive urine cultures, with Wilson 95% confidence intervals. Later estimates are descriptive and imprecise because of the small sample sizes.

**Table 1:** Participant flow through the study.

Stage	Records, *n*
Laboratory records screened (female patients with recorded dysglycaemia, May 2016–September 2025)	126
Excluded: no organism entry at index culture	57
Excluded: documented as no significant growth	2
Included in analysis (index positive culture)	67
Contributed a second positive culture	28
Contributed a third positive culture	10
Total positive urine cultures analysed	105

All consecutive eligible records within the study window were extracted; no subsampling was applied. The 1885 female ICD-10 N39.0 encounters recorded during 2024 (Section 2.1), derived from the hospital information system, cannot be linked at patient level to this laboratory extract and do not constitute the sampling frame.

**Table 2:** Characteristics of the study cohort (*n* = 67).

Characteristic	Value	Completeness
Age, years, mean (SD)	51.6 (17.8)	51/67 (76.1%)
Age, years, median (range)	49 (13–85)	51/67 (76.1%)
HbA1c, %, mean (SD)	8.0 (2.1)	65/67 (97.0%)
HbA1c, %, median (range)	7.5 (5.6–16.3)	65/67 (97.0%)
HbA1c ≥ 7.0%, *n* (%)	40 (61.5)	of 65 measured
HbA1c < 6.5%, *n* (%)	12 (18.5)	of 65 measured
Glycaemic status: diabetes range, *n* (%)	53 (79.1)	of 67
Glycaemic status: prediabetes range, *n* (%)	14 (20.9)	of 67
Second positive culture, *n* (%)	28 (41.8)	95% CI 30.5–53.7
Third positive culture, *n* (%)	10 (14.9)	95% CI 7.9–24.9
Interval, index-to-second culture, days, median (IQR)	161 (74–402)	27/28 (96.4%)
Interval, second-to-third culture, days, median (IQR)	185 (86–392)	9/10 (90.0%)

SD, standard deviation; CI, confidence interval; IQR, interquartile range. Percentages for second and third positive cultures are calculated using the full index cohort (*n* = 67). The completeness column is shown because age was missing for approximately one quarter of participants. Glycaemic status is classified by the operational definition in Section 2.2: the diabetes range comprises HbA1c ≥ 6.5%, and the prediabetes range comprises HbA1c 5.7–6.4%, impaired fasting glucose or impaired glucose tolerance.

**Table 3:** Distribution of organisms across successive positive urine cultures.

Organism	Index Culture (*n* = 67)	Second Culture (*n* = 28)	Third Culture (*n* = 10)
*E. coli*, non-ESBL-producing	26 (38.8)	10 (35.7)	5 (50.0)
*E. coli*, ESBL-producing	17 (25.4)	4 (14.3)	3 (30.0)
*Candida* spp.	13 (19.4)	5 (17.9)	0
*K. pneumoniae*, non-ESBL-producing	4 (6.0)	1 (3.6)	1 (10.0)
*K. pneumoniae*, ESBL-producing	2 (3.0)	1 (3.6)	1 (10.0)
*Enterobacter* spp.	2 (3.0)	1 (3.6)	0
*Pseudomonas aeruginosa*	1 (1.5)	2 (7.1)	0
*Streptococcus agalactiae* (GBS)	0	2 (7.1)	0
*Klebsiella oxytoca*	0	1 (3.6)	0
*Proteus mirabilis*	1 (1.5)	0	0
*Citrobacter koseri*	1 (1.5)	0	0
*Kluyvera ascorbata*	1 (1.5)	0	0
MRSA	1 (1.5)	0	0
Mixed growth/urethral flora (possible contamination)	1 (1.5)	1 (3.6)	0

Values are *n* (%) of specimens. ESBL, extended-spectrum beta-lactamase; GBS, group B *Streptococcus*; MRSA, methicillin-resistant *Staphylococcus aureus*. The index column comprises 65 monomicrobial specimens, one specimen yielding two organisms and one yielding three, giving 70 organism entries from 67 specimens; percentages are calculated on 67 specimens and therefore sum to slightly above 100%. ESBL-producing *E. coli* is shown separately as a resistance phenotype but represents the same species as non-ESBL *E. coli*.

**Table 4:** Recovery of the same organism or resistance-phenotype category at two positive urine cultures in the same participant.

Organism	Cultures 1 and 2 (*n* = 28)	Cultures 2 and 3 (*n* = 10)	Cultures 1 and 3 (*n* = 10)
*E. coli*, non-ESBL-producing	5 (17.9)	1 (10.0)	2 (20.0)
*E. coli*, ESBL-producing	4 (14.3)	2 (20.0)	3 (30.0)
*Candida* spp.	2 (7.1)	0	0

Values are *n* (%). Denominators are the numbers of participants with both cultures in the relevant comparison: 28 for the index-to-second comparison and 10 for comparisons involving the third culture. Category-level recovery does not distinguish persistence or relapse from reinfection without strain typing.

## Data Availability

The fully de-identified dataset supporting the reported findings is provided as Supplementary Materials and is also available from the corresponding author on reasonable request.

## Supplementary Materials

The following supporting information can be downloaded at: https://www.mdpi.com/article/10.3390/microorganisms14102126/s1, Table S1: Fully de-identified participant-level dataset supporting all reported analyses (Microsoft Excel).
